# Case Report: Molecular Characterization of Aggressive Malignant Retroperitoneal Solitary Fibrous Tumor: A Case Study

**DOI:** 10.3389/fonc.2021.736969

**Published:** 2021-12-22

**Authors:** Haruna Nonaka, Shuya Kandori, Satoshi Nitta, Masanobu Shiga, Yoshiyuki Nagumo, Tomokazu Kimura, Takashi Kawahara, Hiromitsu Negoro, Akio Hoshi, Takahiro Kojima, Koji Kawai, Bryan J. Mathis, Takuro Tamura, Taka-Aki Sato, Mariko Yamato, Masayuki Noguchi, Hiroyuki Nishiyama

**Affiliations:** ^1^ Department of Urology, Faculty of Medicine, University of Tsukuba, Tsukuba, Japan; ^2^ Department of Urology, Aichi Cancer Center Hospital, Nagoya, Japan; ^3^ Department of Urology, School of Medicine, International University of Health and Welfare, Narita, Japan; ^4^ International Medical Center, University of Tsukuba Affiliated Hospital, Tsukuba, Japan; ^5^ Research and Development Centre for Precision Medicine, University of Tsukuba, Tsukuba, Japan; ^6^ Department of Pathology, Faculty of Medicine, University of Tsukuba, Tsukuba, Japan

**Keywords:** malignant solitary fibrous tumor, *NAB2-STAT6* fusion gene, STAT6 nuclear expression, target DNA sequencing, *TP53* mutation

## Abstract

Solitary fibrous tumors (SFT) are mesenchymal neoplasms with a favorable prognosis usually originating from the visceral pleura. Rarely, they may occur at various extrapleural sites and show malignant behavior coupled with dedifferentiation. NAB2-STAT6 fusion gene and STAT6 nuclear expression are biomarkers for diagnosis of SFT in addition to CD34, Bcl-2, and CD99. Furthermore, several reports have shown specific NAB2-STAT6 fusion variants and loss of STAT6 protein expression are associated with malignancy. We report a rare case of retroperitoneal SFT which rapidly progressed to death within 35 days after admission. Autopsy found a primary tumor containing both benign and malignant histologies, with multiple metastatic sites similar to the malignant, dedifferentiated tumor. STAT6 was detected in the primary differentiated tumor but not in the primary dedifferentiated tumor or lung/liver metastases. However, the NAB2-STAT6 fusion gene (NAB2ex6/STAT6ex16 variant) was detected in the primary tumor and lung/liver metastases. Intriguingly, fusion gene expression at the transcriptional level was downregulated in the dedifferentiated tumors compared to the differentiated tumor. We further performed target DNA sequencing and found gene mutations in TP53, FLT3, and AR in the dedifferentiated tumors, with TP53 mutations especially found among them. We demonstrate that downregulation of NAB2-STAT6 fusion gene at the transcriptional level is associated with malignant SFT for the first time. Moreover, the present study supports the idea that TP53 mutations promote malignancy in SFTs.

## Introduction

Solitary fibrous tumors (SFT) are mesenchymal neoplasms usually originating from the visceral pleura but can occur at various extrapleural sites (1). They are usually slow-growing with favorable prognoses but approximately 10-20% develop malignancy (2, 3). SFTs are diagnosed by histologic features and CD34 immunostaining while positive findings for Bcl-2 and CD99 are supportive for diagnosis (1). However, recent advances in next generation sequencing have established a fusion gene of juxtaposed NGFI-A binding protein 2 (NAB2) and signal transducer and activator of transcription 6 (STAT6) as the genetic hallmark of SFT (4, 5). Subsequently, immunohistochemical detection of STAT6 nuclear expression is reported as a highly sensitive and specific biomarker for SFT diagnosis (6, 7). Here, we present a rare case of retroperitoneal SFT where the primary tumor contained a clear delineation between benign (CD34- and STAT6-positive) and malignant (CD34- and STAT6-negative) histologies. After diagnosis, the patient quickly died from rapid exacerbation of metastases. Here, we analyze the molecular characteristics of this unique case and discuss mechanisms for the observed malignant phenotype.

## Material and Methods

### Immunohistochemistry

The tissue specimens were cut into 4μm-thick sections from formalin-fixed, paraffin-embedded (FFPE) blocks before deparaffinization and antigen retrieval using PT Link (Dako, Agilent Technologies). Target retrieval solution ‘high’ was used for staining of CD34 and STAT6 while ‘low’ was used for Ki67. Immunostaining was performed using a Dako Autostainer Link 48 (Agilent Technologies) with the primary antibody (anti-Ki67 antibody [Cat#:IR626, Dako], anti-CD34 antibody [Cat#:IR632, Dako] or anti-STAT6 antibody [1:400 dilution, Cat#: SC-621, SantaCruz]) and REAL Envision HRP rabbit/mouse (Agilent Technologies) as a secondary antibody. Immunoreactivity was detected with DAB (Dako REAL EnVision Detection system, Agilent Technologies) and counterstaining was performed with hematoxylin.

### RT-PCR and Sanger Sequencing

Total RNA was extracted from frozen samples by RNeasy Mini Kit (Qiagen). The RNA was then reverse-transcribed into cDNA using a High Capacity cDNA Reverse Transcription Kit (Thermo Fisher Scientific). PCR was performed by Quick Taq^®^ HS DyeMix (TOYOBO) according to the manufacturer’s instructions. The primer sets for the detection of NAB2-STAT6 fusion genes were previously designed by Tai et al. (8). Hypoxanthine phosphoribosyltransferase 1 (HPRT) was used as an internal control. Primer sequences are listed in **Supplementary Table 1**. PCR products were loaded onto 2% agarose gels with ethidium bromide and visualized under UV illumination. Confirmed PCR products were directly sequenced using an Applied Biosystems 3730xl Genetic Analyzer (Thermo Fisher Scientific).

### Next-Generation Sequencing

DNA was extracted from frozen samples by QIAamp DNA Mini Kit (Qiagen) or from FFPE samples by AllPrep DNA/RNA FFPE kit (Qiagen). A QIAseq Human Comprehensive Cancer Panel (DHS-3501Z-12, Qiagen) was used for library construction according to the manufacturer’s instructions. The libraries were assessed using a Bioanalyzer High Sensitivity DNA Kit (5067-4626, Agilent Technologies) and applied to a MiSeq sequencer (Illumina) to obtain 2x151-base reads. FASTQ files were imported to CLC Genomics Workbench (ver.12.0, Qiagen) and compared with normal kidney to remove the germline mutations. Somatic mutations were selected by allele frequency >=5% and coverage >=100x.

## Case Presentation

### Clinical Summary

**Figure 1:**
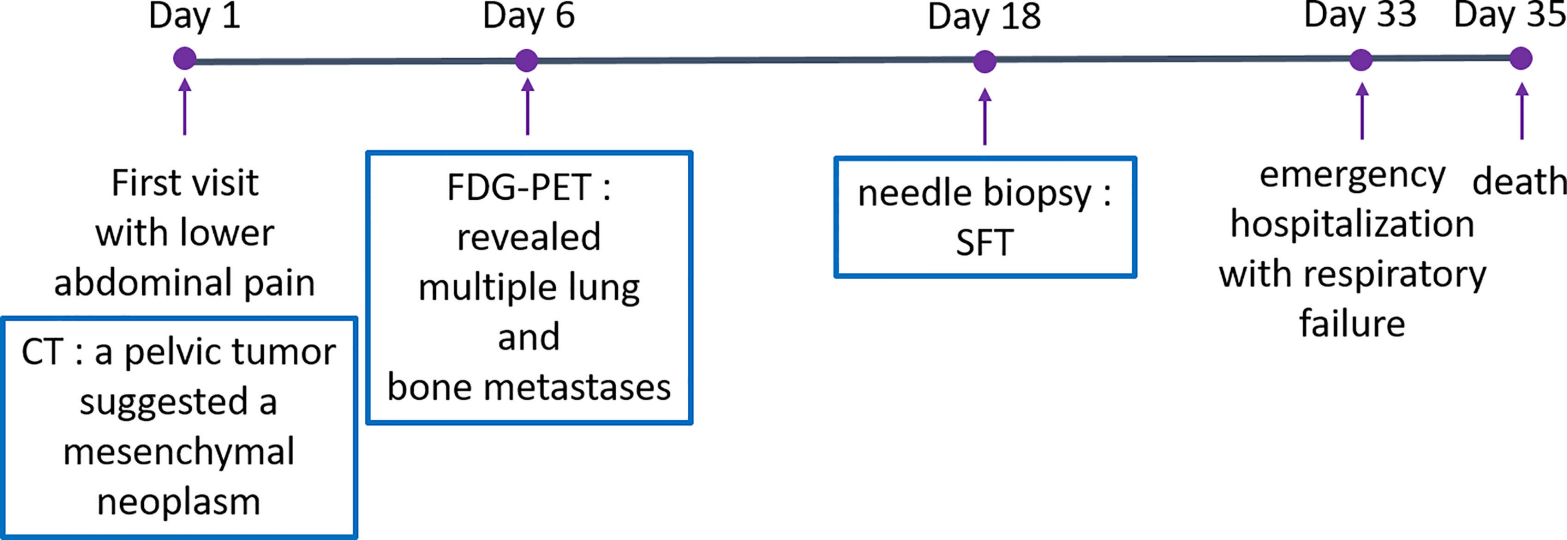
Case report timeline.

**Figure 2:**
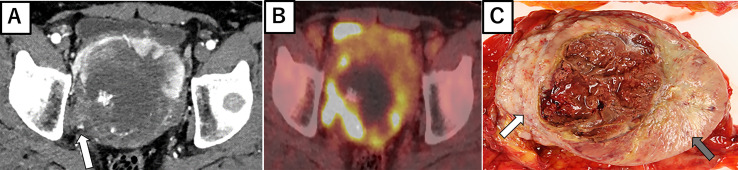
Radiological imaging and macroscopic findings of primary site. **(A)** Enhanced abdominal CT showing central necrosis and the marked enhancement of anterior to left periphery of the tumor. At the right periphery, the tumor is seen invading the pelvic wall (white arrow). **(B)** 18F-fluorodeoxyglucose-positron emission tomography showing the marked hypermetabolism in the right invasive area of the tumor. **(C)** Autopsy specimen, with a well-circumscribed and smooth area (left side: gray arrow) and poorly margined and lobulated area (right side: white arrow).

A 53-year-old man with an unremarkable past medical history presented to our department with lower abdominal pain. Enhanced chest and abdominal computed tomography (CT) showed a 10x10x10 cm pelvic tumor and, although central necrosis was revealed, the anterior-to-left periphery of the tumor was markedly enhanced. The tumor margin was clear in most parts but had partly invaded into the right pelvic wall. Marked hypermetabolism in the invasive area of the tumor was seen on 18F-fluorodeoxyglucose-positron emission tomography (FDG PET) (**Figures 2A, B**) and both CT and FDG PET revealed multiple lung and bone metastases. We clinically diagnosed retroperitoneal sarcoma or malignant mesenchymal tumors based on the image findings. Therefore, we next carried out percutaneous needle biopsy of the primary masses to determine a pathological diagnosis, finding specimens composed of spindle-shaped malignant cells positive for STAT6 that led to a pathological diagnosis of solitary fibrous tumor (SFT). Cytotoxic chemotherapy was planned but the patient’s condition rapidly deteriorated, with respiratory failure, disseminated intravascular coagulation, and finally death from multiple organ failure occurring 35 days after admission. A chronological summary of the case report is shown in **Figure 1**.

### Autopsy Findings

**Figure 3:**
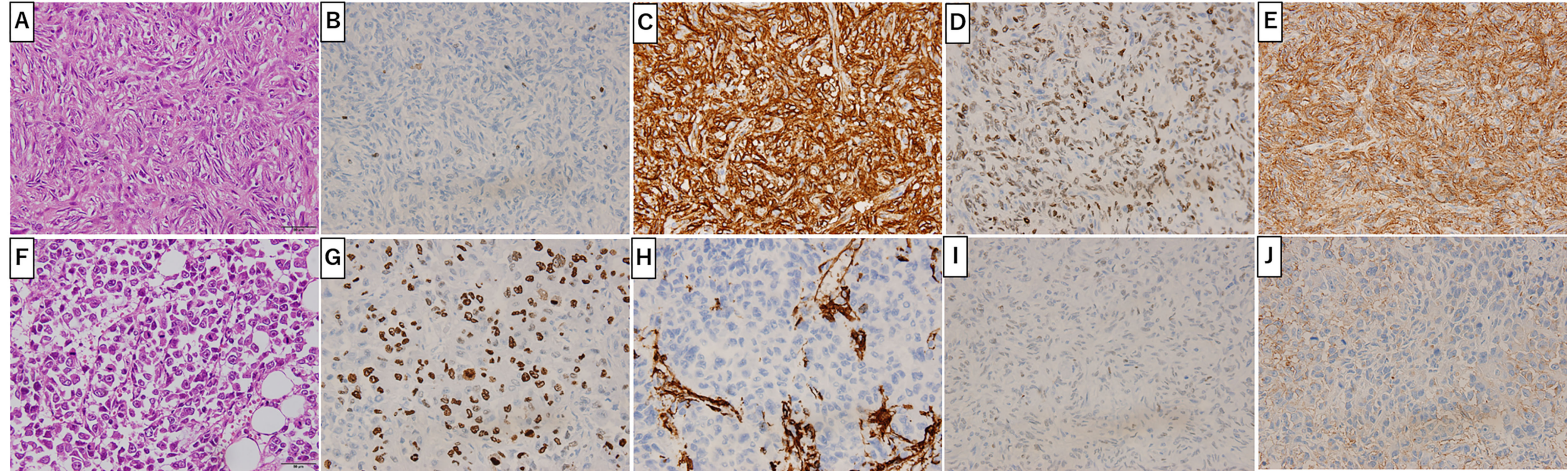
Primary Site Pathology. Pathologic findings were distinct between the left **(A-E)** and right **(F-J)** sides of the tumor. On the left side (gray arrow in **Figure 2C**), spindle-shaped tumor cells with mild atypia and collagen fiber proliferation are seen **(A)**. Ki-67 positive cells were around 1% **(B)**. Tumor cells positively stained with CD34 **(C)**, STAT6 **(D)** and CD99 **(E)**. On the right side (white arrow in **Figure 2C**), round or short spindle-shaped tumor cells with high N/C ratios proliferating in a honeycomb pattern are seen **(F)**. Around 80% of tumor cells were positive for Ki-67 **(G)** and CD99 **(J)** but not CD34 **(H)** or STAT6 **(I)**. Scale bar: 50µm.

**Figure 2C** shows the gross findings from the tumor autopsy, namely a well-circumscribed and smooth area on the left side and a poorly margined and lobulated area on the right side corresponding to radiological imaging findings (**Figures 2A, B**). Pathological findings also differed between the left (**Figures 3A–E**) and right sides (**Figures 3F–J**) of the tumor. On the left side, spindle-shaped tumor cells with mild atypia and collagen fiber proliferation were observed. The mitotic activity was 0.3/10 HPF and tumor necrosis was not observed. The Ki-67-positive rate was low, around 1% (**Figure 3B**), but cells were diffusely stained with CD34 (**Figure 3C**) and tumor cell nuclei were positively stained with STAT6 (**Figure 3D**). The left side findings fit the definition of SFT in the WHO Classification (9). On the right side, round or short spindle-shaped tumor cells with high N/C ratios proliferated in a honeycomb pattern. In contrast to the left side, the mitotic activity was 80-100/10 HPF and tumor necrosis was detected. Moreover, the around 80% of these tumor cells were stained with Ki-67 (**Figure 3G**) but completely negative for CD34 and STAT6 (**Figures 3H, I**). Tumor cells from both sides were positive for CD99 (**Figures 3E, J**), focally positive for BCL-2, and negative for p53 (data not shown). From these observations, the left side lesion was considered to be the differentiated SFT while the right side lesion was composed of a dedifferentiated tumor. We next conducted a complete histological examination of the multiple metastatic sites revealed at autopsy, including more than 30 lung metastases, 2 liver metastases, 3 bone metastases and 1 adrenal metastasis, and pathological findings for all sites were similar to the right-side dedifferentiated tumor. We thus diagnosed the dedifferentiated tumors as malignant SFT, which were transformed from the differentiated SFT on the left side of the primary site.

### Molecular Findings

**Figure 4:**
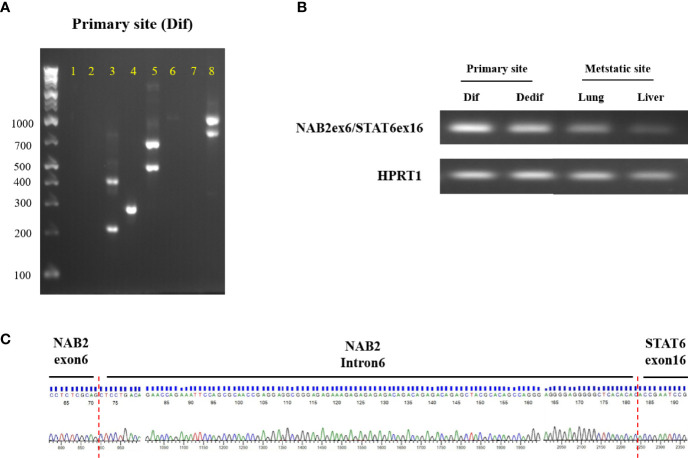
NAB2-STAT6 fusion gene status in primary and metastatic sites. **(A)** Agarose gel separation of a NAB2-STAT6 fusion-specific RT-PCR product (NAB2ex6/STAT6ex16, Lane 4) from the primary differentiated tumor. **(B)** Detection of NAB2ex6/STAT6ex16 fusion gene from the primary site (differentiated and dedifferentiated tumors) and metastatic sites. **(C)** Sanger sequencing chromatogram of a NAB2/STAT6 fusion-specific RT-PCR product. Dif: differentiated tumor. Dedif: dedifferentiated tumor.

Detecting the NAB2/STAT6 fusion gene is the gold standard for SFT diagnosis but, in the present case, STAT6 protein expression within dedifferentiated tumors was not observed. To clarify NAB2/STAT6 fusion gene status, we performed RT-PCT analysis of the primary differentiated tumor, primary dedifferentiated tumor, and lung/liver metastases. The NAB2 exon 6/STAT6 exon 16 (NAB2ex6/STAT6ex16) variant was identified in the primary differentiated tumor (**Figure 4A**), with Sanger sequencing revealing a stretch (111bp) of NAB2 intronic sequence between the NAB2 exon 6 and STAT6 exon 16 (**Figure 4B**). The NAB2ex6/STAT6ex16 variant was also detected in the primary dedifferentiated tumor and lung/liver metastases (**Figure 4C**), albeit at lower levels compared to the primary differentiated tumor.

**Figure 5:**
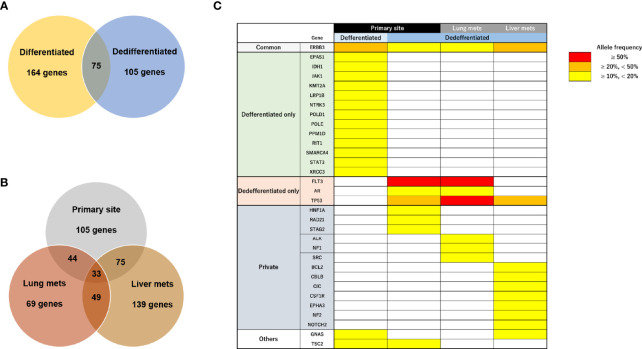
Characteristics of genomic alterations in primary and metastatic sites. **(A)** Number of all gene mutations between primary differentiated and dedifferentiated tumors. **(B)** Number of all gene mutations between primary and metastatic sites in dedifferentiated tumors. **(C)** Mutational heatmap for the primary differentiated tumor, primary dedifferentiated tumor, and lung/liver metastases. Mutations with variant allele frequency <10% were excluded.

We next performed target DNA sequencing using a comprehensive cancer panel for the primary differentiated tumor, primary dedifferentiated tumor, and lung/liver metastases. All non-synonymous variants are listed in **Supplementary Tables 2-5**. The number of gene mutations was 164 in the primary differentiated tumor, 105 in the primary dedifferentiated tumor, 69 in the lung metastasis and 139 in the liver metastasis (**Figures 5A, B**). At the primary site, 75 genes were shared between the differentiated and dedifferentiated tumors. In the dedifferentiated tumors, 33 genes were shared between the primary and metastatic sites. Oncoprinting of gene mutations among four lesions is shown in **Supplementary Table 6**. Mutations with variant allele frequency <10% were excluded from further analysis to clarify the significance of the mutations and a summary is shown in **Figure 5C**. The gene mutations in TP53, FLT3, and AR were found in dedifferentiated tumors while the TP53 mutation (c.97del; p.Ser33fs) was especially found in all dedifferentiated tumors.

## Discussion and Conclusions

Here, we present a rare case of retroperitoneal SFT with clear delineation between benign and malignant histologies within a single primary tumor. The malignant side met the criteria for a pathological judgement of malignancy, namely high cellularity and mitotic activity (more than four mitotic figures per 10 high-power fields), pleomorphism, hemorrhage, and necrosis (10). Pathological findings of all metastatic sites obtained at autopsy were similar to the malignant, dedifferentiated lesion. Demicco et al. reported a risk stratification model for SFT incorporating patient age, tumor size, mitotic activity and necrosis to predict metastatic risk (11). According to this model, no metastases were seen in low risk cases, while intermediate risk patients had a 10% risk of metastasis at 10 years and high-risk SFT had a 73% risk of metastasis at 5 years. The present case was categorized as intermediate risk; nevertheless, the disease rapidly progressed and patient condition quickly deteriorated. This suggests that clinicopathological classification has limits to its prognostic power and molecular characterization may be more precise in this regard.

In the present case, we planned a conventional chemotherapy regimen but the rapid death precluded any treatment, highlighting the fact that systemic therapeutic options for unresectable or metastatic SFT disease are particularly limited. Anthracycline-based regimens have been advocated as a first-line chemotherapy, with several retrospective studies showing that the median progression-free survival (PFS) was 3-5 months in such patients with advanced SFT (12). On the other hand, some anti-angiogenic agents, such as sunitinib or pazopanib, have demonstrated efficacy for patients with advanced SFT and the median PFS was 4.7-9.7 months in those studies (12). Although a number of therapeutic agents have been tested in patients with advanced SFT, the efficacy of systemic therapies is limited. Therefore, an approach based on molecular mechanisms, particularly those driving tumorigenesis or progression of SFT, might pave the way to new therapeutic strategies.

With regard to these molecular strategies, NAB2-STAT6 fusion, recognized as a hallmark of SFT, was first identified by Robinson et al. and Chmielecki et al. from whole-exome sequencing studies (4, 5). These fusion genes drive STAT6 nuclear expression and immunohistochemical detection has been generally recognized as a definitive biomarker for diagnosis of SFT. A meta-analysis revealed that more than 40 NAB2-STAT6 fusion variant types were present in up to 83% (452/546) of SFTs, with NAB2ex6-STAT6ex16/17/18 and NAB2ex4-STAT6ex2/3 being the most frequent variants (13). In the present case, the NAB2ex6–STAT6ex16 variant was identified in primary and metastatic sites by RT-PCR and Sanger sequencing (**Figure 4**). Barthelmess et al. reported that NAB2ex6–STAT6ex16/17 was significantly associated with malignant phenotype and high recurrence in SFTs (14). Similarly, Akaike et al. showed that SFTs with NAB2ex6–STAT6ex16/18 harbored aggressive histological features (15). However, several studies have reported that NAB2-STAT6 fusion variants were not associated with malignant SFTs (16–19) and, therefore, the association between NAB2-STAT6 fusion variants and malignant potential remains controversial (14–19). These findings suggest that some other molecular mechanism promoted the malignant transformation seen in the present case.

Several studies reported that 7% to 10% of SFTs are negative for CD34 (6, 16) while, in contrast, STAT6 is highly positive in SFTs. Tai et al. reported that 87 of 88 (99%) tumors diagnosed as SFT (75 nonmalignant and 13 malignant) were positive for STAT6 (8) but STAT6 was positive in 7 of 8 CD34-negative SFTs. Other studies also reported that positive rates of STAT6 in SFTs were from 98% to 100% (6, 7). On the other hand, Dagrada et al. showed that STAT6 protein expression in dedifferentiated recurrent/metastatic tumors was lost whereas expression in primary usual/malignant tumors was positive in 4 SFT cases (16). RT-PCT analysis found the NAB2-STAT6 fusion gene in 3 of 4 cases in that report (16). Zhang et al. also reported a case with mediastinal malignant SFT carrying a NAB2-STAT6 fusion gene but negative STAT6 immunohistochemical staining (20). In the present case, the primary differentiated tumor was positive for CD34 and STAT6 but the primary dedifferentiated tumor and lung/liver metastases were completely negative (**Figures 3C, D, H, I**). Moreover, NAB2ex6-STAT6ex16 fusion gene expression was downregulated in the dedifferentiated tumors compared to the differentiated tumor (**Figure 4B**). These findings suggest that NAB2-STAT6 chimeric protein downregulation promotes malignant transformation and might involve some transcriptional mechanism.

Previous reports have shown that other molecular factors, such as mutations in TP53, TERT promoter, and APAF1, were associated with malignant transformation or dedifferentiation (15–17, 21–24). Park et al. demonstrated that TP53 immunohistochemical positive in SFTs was significantly associated with malignant cases and TP53 mutations were detected in 41% of malignant SFTs (17). Similarly, TP53 mutations were detected only in dedifferentiated tumors in the present case. The identified mutation (c.97del in TP53) was previously found in patients with head and neck or esophageal squamous cell carcinoma (25, 26). Although this mutation has been linked to malignant transformation or dedifferentiation of SFT (15–17, 22–24), the mechanisms remain to be fully elucidated. On the other hand, FLT3 mutations (c.20A>G; p.Asp7Gly) were detected in the primary dedifferentiated tumor and lung metastases of the present case with high allele frequencies (77.6% and 93.1%), contributing to respiratory failure due to rapid progression of multiple lung metastases. As FLT3 mutations frequently occur in acute myeloid leukemia (AML) (27) and are associated with transformation to AML in myelodysplastic syndrome (MDS) patients (28, 29), these findings led us to speculate that an FLT3 mutation also promoted the malignant transformation of the dedifferentiated tumors in the present case.

In conclusion, we experienced a rare case of retroperitoneal SFT with rapid and lethal progression. Interestingly, two morphologic tumor types (differentiated and dedifferentiated) coexisted at the primary site but metastatic sites contained dedifferentiated tumors that commonly featured loss of STAT6 protein and TP53 mutations. Here, we demonstrate that downregulation of the NAB2-STAT6 fusion gene at the transcriptional level is associated with malignant SFT for the first time. These findings suggest that specific molecular alterations are associated with malignant behaviors, indicating that vigilance is required against SFT cases with loss of STAT6 protein expression and TP53 mutations.

## Data Availability Statement

The datasets of this study have been deposited with links to BioProject accession number PRJDB11977 in the DDBJ BioProject database. 

## Ethics Statement 

This study was reviewed and approved by the Ethics Committee of the University of Tsukuba (Approval Number: H28-104). The written informed consent was obtained from the bereaved families of the patient at the time of autopsy.

## Author Contributions

HNo: data collection and manuscript writing. SK: conception and manuscript writing. revising. SN, MS, TKi, TKa, and AH: data collection. YN, MY, and TT: data analysis and interpretation. HNe, TKo, KK, and BM: manuscript revising. T-AS, MN, and HNi: supervision. All authors have read and agreed to the published version of the manuscript. All authors contributed to the article and approved the submitted version.

## Funding

This work was supported by a grant from COI-NEXT (JPMJPF2017).

## Conflict of Interest

The authors declare that the research was conducted in the absence of any commercial or financial relationships that could be construed as a potential conflict of interest.

## Publisher’s Note

All claims expressed in this article are solely those of the authors and do not necessarily represent those of their affiliated organizations, or those of the publisher, the editors and the reviewers. Any product that may be evaluated in this article, or claim that may be made by its manufacturer, is not guaranteed or endorsed by the publisher.
